# Exact comprehensive equations for the photon management properties of silicon nanowire

**DOI:** 10.1038/srep24847

**Published:** 2016-04-22

**Authors:** Yingfeng Li, Meicheng Li, Ruike Li, Pengfei Fu, Tai Wang, Younan Luo, Joseph Michel Mbengue, Mwenya Trevor

**Affiliations:** 1 State Key Laboratory of Alternate Electrical Power System with Renewable Energy Sources, North China Electric Power University, Beijing, 102206, China; 2 Chongqing Materials Research Institute, Chongqing, 400707, China

## Abstract

Unique photon management (PM) properties of silicon nanowire (SiNW) make it an attractive building block for a host of nanowire photonic devices including photodetectors, chemical and gas sensors, waveguides, optical switches, solar cells, and lasers. However, the lack of efficient equations for the quantitative estimation of the SiNW’s PM properties limits the rational design of such devices. Herein, we establish comprehensive equations to evaluate several important performance features for the PM properties of SiNW, based on theoretical simulations. Firstly, the relationships between the resonant wavelengths (RW), where SiNW can harvest light most effectively, and the size of SiNW are formulized. Then, equations for the light-harvesting efficiency at RW, which determines the single-frequency performance limit of SiNW-based photonic devices, are established. Finally, equations for the light-harvesting efficiency of SiNW in full-spectrum, which are of great significance in photovoltaics, are established. Furthermore, using these equations, we have derived four extra formulas to estimate the optimal size of SiNW in light-harvesting. These equations can reproduce majority of the reported experimental and theoretical results with only ~5% error deviations. Our study fills up a gap in quantitatively predicting the SiNW’s PM properties, which will contribute significantly to its practical applications.

High-performance photon management (PM) at nanoscale dimensions is important for many future optical and optoelectronic devices^1^,^2^,^3^,^4^,^5^. Due to the great importance of silicon in optical and optoelectronic fields, silicon resonant nanostructures especially silicon nanowire (SiNW) attracts special attentions for PM^6^,^7^,^8^,^9^,^10^,^11^,^12^,^13^,^14^,^15^. Many experimental and theoretical works have demonstrated that SiNW is of excellent collection ability for light of certain wavelengths: it can capture light in an area 100 times of its geometrical cross section^5^,^16^. This makes SiNW have wider applications in photodetectors^17^,^18^,^19^, optical sensor^20^,^21^,^22^,^23^,^24^,^25^, and photovoltaics^26^,^27^,^28^,^29^,^30^. Furthermore, because of the high refraction index of silicon material, the captured light by the SiNW is mostly confined within itself 31,32. This makes SiNW promising in nanowire waveguides and switches^33^,^34^,^35^,^36^.

The PM functions of SiNW have been successfully attributed to the optical coupling between the incident light and the leaky modes supported by the nanowire^6^,^17^,^37^. Under this theoretical framework, the most effective optical coupling occurs at some certain wavelengths, usually called the resonant wavelength (RW), where SiNW can function in light-harvesting most effectively. The existence of RW results in that the performance of some SiNW-based photonic devices (like photodetectors^17^,^18^,^19^ and optical sensors^20^,^21^,^22^,^23^,^24^,^25^), and the optical interconnect efficiency between adjacent SiNW waveguides^33^,^34^,^35^,^36^ have remarkable wavelength selectivity. In some sense, RW is the most important performance parameter for the PM properties of SiNW. However, to the best of our knowledge, although it has been made clear that RW is of strong and weak dependences on the diameter and length of SiNW respectively^17^,^37^,^38^,^39^, there is still no efficient equations to describe such dependencies quantitatively.

As mentioned above, SiNW possesses the maximum light-harvesting efficiency at RW (LHE-R). This means LHE-R determines the sensitivity limit of SiNW-based photodetectors and optical sensors^17^,^18^,^19^,^20^,^21^,^22^,^23^,^24^,^25^, and will have direct impact on the insertion loss in optical interconnects^33^,^35^ as well as optical switches^34^. Hence LHE-R should be the second important feature for SiNW’s PM properties. It has been found that the SiNW’s absorption cross-section at RW can reach 1–2 orders of magnitude higher than its geometry cross sectional area^5^,^16^. However, there is still no report on studying the quantitative relationship between LHE-R and the size of SiNW.

For the applications of SiNW in photovoltaic devices, like solar cells^26^,^28^,^30^, sunlight-driven solar water splitting devices^12^,^14^ and photoelectrochemical cell^27^,^29^, the device performances should dramatically depend on the SiNW’s light-harvesting efficiency in full-spectrum (LHE-F). As being reported^37^, a 300% photocurrent enhancement can be achieved in single SiNW-based solar cell compared with the bulk silicon ones. So, LHE-F has special significance for photovoltaic devices, thus should be another critical performance feature for SiNW’s PM properties. Nevertheless, to date there is also none of quantitative study on the size dependency of LHE-F. The lack of quantitative researches on the PM properties of SiNW will greatly limit the rational design of SiNW-based photonics devices.

In this work, based on discrete dipole approximation (DDA) simulations^40^, we systematically investigate RW, LHE-R and LHE-F of SiNW, and establish exact comprehensive equations to describe their quantitative relationships with the diameter and length of SiNW. The reliability and practicability of these equations have been verified. Furthermore, using these equations, four extra equations are deduced to estimate the optimal size of SiNW in light-harvesting. This work is of great help for the applications of SiNW in future photonic devices.

## Results and Discussion

**Figure 1:**
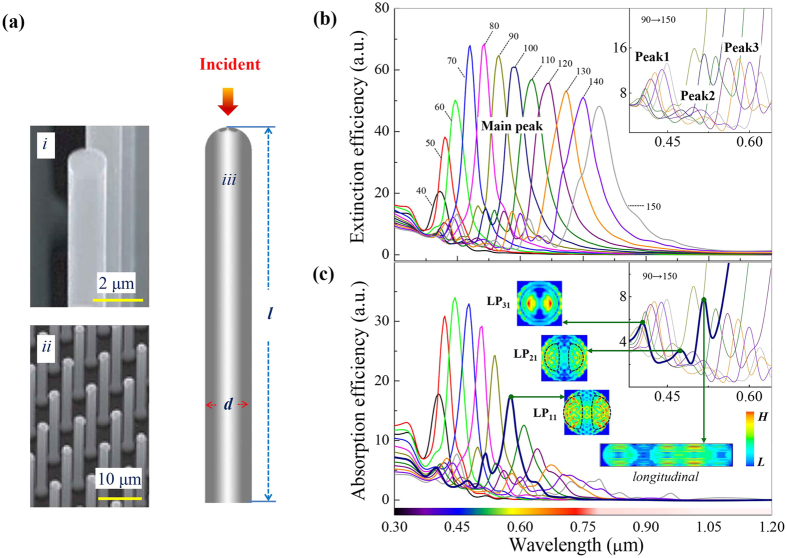
Model and the PM properties of SiNW. (**a**) SEM images of SiNW from ref. 41 (i) and ref. 16 (ii), and the model used in this study (iii). (**b**) Extinction and (**c**) absorption efficiency curves of SiNW with fixed length (0.5 μm) but various diameters (40–150 nm). The insets in (**c**) are the electric filed distribution in SiNW of 100 nm diameter, at different peaks.

We model SiNW as a circular cylinder with hemispherical tip, as shown in Fig. 1a, referring to the structures fabricated by the V-L-S technology^16^,^41^. The length *l* and diameter *d* range from 0.5 to 10 μm and from 30 to 200 nm, respectively, and the PM properties are simulated using DDSCAT 7.3^40^, whose reliability has been fully verified^42^,^43^,^44^,^45^. Since the PM properties of SiNW is polarization-independent^6^,^39^ and weak angle-dependent^37^,^46^, only the linearly polarized incident light illuminating from the tip (corresponding to incident angle θ = 0°) is considered. Bulk values of the complex index of refraction for silicon are used^47^.

The PM properties of SiNW are characterized by the extinction and absorption efficiencies, see Fig. 1b,c, which are defined as *Q*_*ext*_ = *C*_*ext*_/*πr*^2^ and *Q*_*abs*_ = *C*_*abs*_/*πr*^2^, respectively. *C*_*ext*_, *C*_*abs*_, and *πr*^2^ denote the extinction, absorption and real geometric cross section of the SiNW respectively. It can be seen that there are four kinds of peaks, which are marked as Main peak, Peak1, Peak2 and Peak3 for convenience. According to the leaky mode theory, these peaks will come from four resonant modes supported by SiNW. We assign mode index them by comparing their wavelengths with the cut-off wavelengths of every possible mode, by taking SiNW of 100 nm diameter as example (given in the Supplementary information). As result, Main peak corresponds to the lowest mode, LP11; while Peak1 and Peak2 correspond to higher order modes LP21 and LP31, respectively. Their electric field distributions are given in the insets of Fig. 1c. Peak3 could not be assigned as its wavelength did no match any mode, but comes from the resonance in the length direction resulting from the reflections at the end-facet^48^.

The curves in Fig. 1b,c reflect the wavelength-selective light-concentration and light-absorption abilities of SiNW. Obviously, SiNW shows the most effective light-harvesting function at RW, i.e. the peaks. Therefore, RW has a direct impact on the single wavelength performance of SiNW-based devices like photodetectors^17^,^18^,^19^, sensors^20^,^21^,^22^,^23^,^24^,^25^ and others^33^,^34^,^35^,^36^; and when use SiNW to trap light, for example in photovoltaics, its RW is also required to locate at the waveband corresponding to the maximum solar irradiance^49^ to collect as much light as possible. In some way, RW is the most important performance feature for the PM properties of SiNW. So, to avoid tedious trial-and-error procedure in designing SiNW-based devices with desired optical resonance, exact quantitative relationships of RW with the size of SiNW are of great necessity.

**Figure 2:**
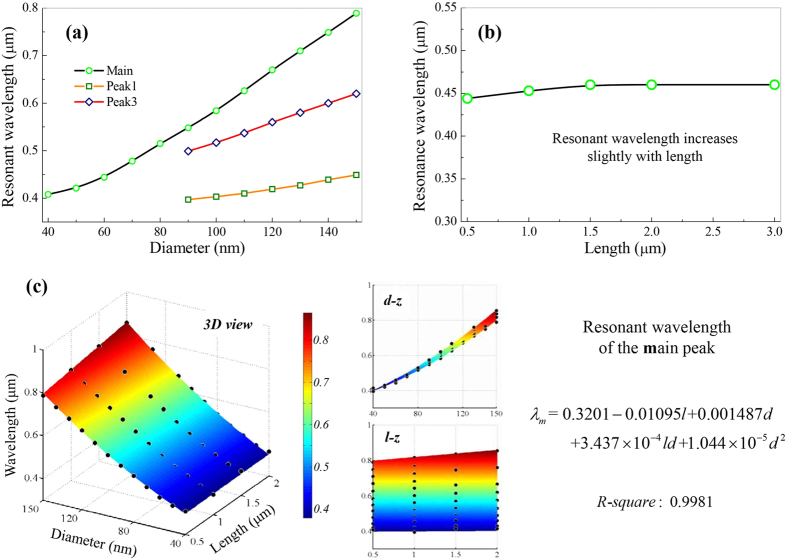
Size dependencies for RW and numerical fitting to obtain the equation at Main peak. (**a**) Diameter decency of RW for SiNW with fixed length 0.5 μm. (**b**) length decency of RW for SiNW with fixed diameter 80 nm. (**c**) The original data (black spheres) and fitted surface (mapped by rainbow) of RW at Main peak, where the *3D* view and the *d-z*, *l-z* side views are given. The fitted polynomial with *R-square* is provided aside.

To accomplish this goal, firstly, the dependency of RW on diameter and length is made clear. By extracting the data from Fig. 1, dependency of RW on diameter is plotted in Fig. 2a, where RW of SiNW, at the Main peak, Peak1 and Peak3, approximately increases linearly with diameter. This dependency is consistent with that of the cut-off wavelength, which can generally reflect RW according to the leaky mode theory (shown in the Supplementary information). The cut-off wavelength can be calculated by the equation 
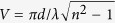
, where *V* is the cut-off parameter, which can be looked-up for every mode; *d* is the diameter of nanowire and *n* is the refraction index of the nanowire material. For specific mode, if *n* is nearly constant, the cut-off wavelength calculated by this equation will be nearly linear diameter dependent. Figure 2b shows the length dependency of RW, based on the extinction and absorption efficiencies of SiNW with fixed diameter (80 nm) and various lengths (0.5–3 μm). Obviously, RW is weakly length dependent. This is because the length of the SiNW is finite, thus the light within the nanowire is also limited in the length direction especially in the nanowire of small draw ratio. It is worth mentioning that, here and hereinafter, Peak2 is not taken into account due to its very weak intensity.

Then, the function forms to express the quantitative relationships are chosen: polynomials are used since the RW at Main peak, Peak1 and Peak3 (symbolized by *λ*_*m*_*, λ*_*p1*_and *λ*_*p3*_) all depend simply on size. Taking *λ*_*m*_as example, considering its linear (but not perfect) diameter and weak length dependencies, we choose its function form as quadratic of *d* and linear of *l*. That is to say, 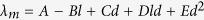, where *A-E* are undetermined coefficients.

Finally, the coefficients are determined by regression approach with the help of the fitting tools in Matlab R2010a (The MathWorks Company), based on a large sample of SiNW with diameter and length ranges from 30 to 150 nm and 0.5 to 2.0 μm respectively. The regression results for *λ*_*m*_ are given in Fig. 2c. The obtained fitting equation is


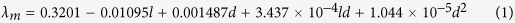


Equations for RW at Peak1 and Peak3 are obtained with the similar process, which are given in the Supplementary Information. The obtained results are listed in Table 1.

How successful the fit is in explaining the variation of the data is quantitatively measured by the correlation index *R-square*, defined as


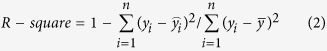


where *i* = *1* − *n* denotes the index of the original data, *y*_*i*_ is the value of the original data, 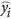 is the corresponding predicted value, and 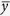 is the average value of the original data. For *λ*_*m*_, the correlation index *R-square* is 0.9981, which means this equation explains 99.81% of the total variation in the original data about the average.

Light-harvesting efficiency (LHE) contains two aspects: light-concentration efficiency (LCE) and light-absorption efficiency (LAE). They refer to the amount of light that can be collected by the nanowire and absorbed within it respectively, and are both of great importance in device application. For example, when nanowire is used in photodetectors^17^,^18^,^19^ or optical sensors^20^,^21^,^22^,^23^,^24^,^25^, the former enables the realization of visible detection, while the latter makes the detection measurable by being transformed to photocurrent^18^. Since the maximum LHE occurs at RW, here, an attempt is made to establish a group of equations to express the quantitative relationship between LHE-R and the size of SiNW. LCE and LAE at RW are characterized by the peak intensities in the extinction and absorption curves, respectively.

**Figure 3:**
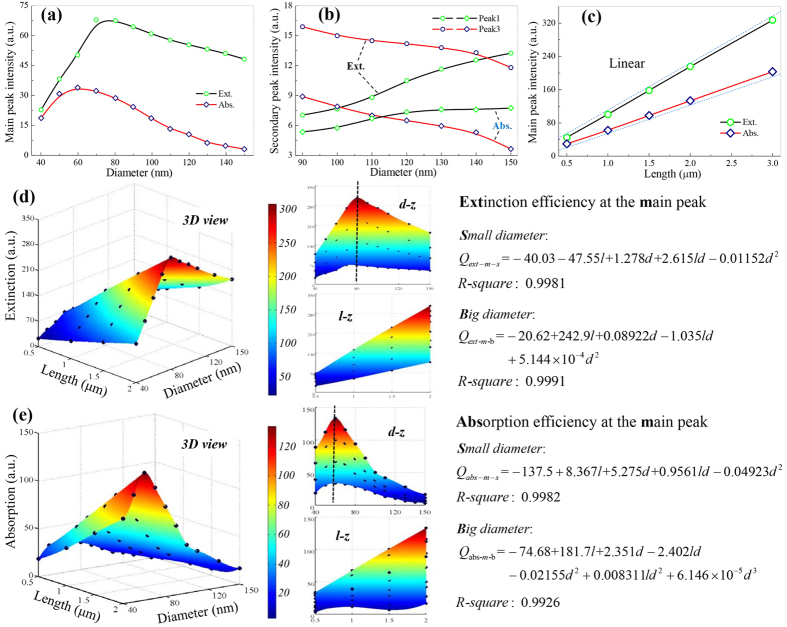
Size dependencies for LHE-R and numerical fitting of its equations at Main peak. Diameter dependency (**a**) at Main peak and (**b**) Peak1, Peak3, for SiNW with fixed length 0.5 μm; (**c**) length dependency, for SiNW with fixed diameter 80 nm. Original data and interpolated surface (rainbow) of the (**d**) extinction and (**e**) absorption intensities at Main peak. The dash lines denote the optimal size of SiNW for light-harvesting. The equations with *R-squares* are given aside.

From Fig. 3a, the diameter dependency of LHE at Main peak, it can be seen that both LCE and LAE initially increase and then decrease with diameter, following a linear trend. Such turning points denote the optimal SiNW size in light-harvesting, which is quantitatively described below. The appearance of the turning points can be attributed to that, if the diameter of SiNW exceeds some value it can supported high-order leaky modes, which correspond to the high-order peaks in Fig. 1b,c. Figure 3b shows the diameter dependency of LHE at Peak1 and Peak3, where the extinction and absorption intensities both show good (but not perfect) linear variation trend with diameter. Both LCE and LAE are of perfect linear length dependency, as illustrated in Fig. 3c. Since the analytic formula of the Mie theory can not be simplified to a simple function of diameter and length, the linear dependencies of LHE on the diameter and length of SiNW is still not clear. However, these dependencies denote that the extinction (absorption) efficiency of SiNW under top illumination is proportional to its longitudinal section area.

We use *Q*_*ext-m-s*_ and *Q*_*ext-m-b*_ (*Q*_*abs-m-s*_ and *Q*_*abs-m-b*_) to describe LCE (LAE) of SiNW, at Main peak, with small and big diameter. According to above analyses, they both show good (but not) linear diameter and perfect linear length dependencies. So, they are set as quadratic of diameter (cubic for *Q*_*abs-m-b*_ to obtain good fitting), and linear of length. *Q*_*ext-p1*_and *Q*_*abs-p1*_are used at Peak1, and *Q*_*ext-p3*_ and *Q*_*abs-p3*_ are used at Peak3. According to their good (but not) linear diameter and perfect linear length dependencies, they are also set as quadratic of diameter (cubic for *Q*_*abs-p1*_ to obtain good fitting), and linear of length.

Taking LCE and LAE of SiNW at Main peak as an example, the fittings are illustrated in Fig. 3d,e respectively. The polynomial functions obtained, adding R-squares are provided aside the figures. The *R-squares*, 0.9981, 0.9991, 0.9982 and 0.9926, show the equations can give perfect fittings of the original data. The equations for LHE of SiNW at Peak1 and Peak3, obtained with the similar process (Supplementary Information) are listed in Table 1.

Since LHE-R at Main peak initially increases and then decreases with diameter, there appear a ridge in Fig. 3d,e respectively. These ridges mean that, SiNW with fixed length has an optimal diameter to get the maximum LHE-R. They are plotted by dash lines on the *d-z* side views as a guide. By letting *Q*_*ext-m-s*_equal to *Q*_*ext-m-b*_, an equation denoting the optimal SiNW size in light concentration is deduced to be


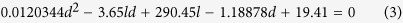


Similarly, the equation denoting the optimal SiNW size in light absorption is


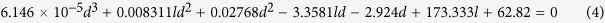


If the SiNW’s lengths are set as 0.5, using these equations, the optimal SiNW diameters for light concentration can be conveniently estimated to be 80.5, 79.9, 79.9 and 79.8 nm; and those for light absorption are 61.4, 61.7, 61.4 and 61.5 nm.

**Figure 4:**
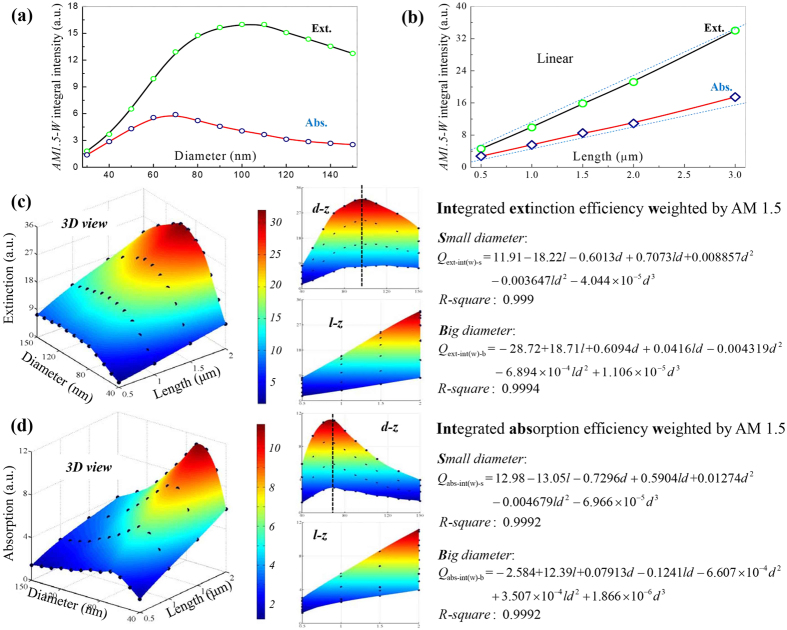
Size dependencies for LHE-F of SiNW and numerical fittings to obtain the equations. (**a**) diameter dependency for SiNW with fixed length 0.5 μm; (**b**) length dependency for SiNW with fixed diameter 80 nm; original data and interpolated surface (rainbow) of the integrated (**c**) extinction and (**d**) absorption intensities, where the dash lines denote the optimal SiNW size. Fitted polynomials with R-square are given aside.

LHE-F is of special significance for photovoltaic devices, whose photocurrent is greatly determined by the amount of light be captured^1^,^4^,^16^. It has been demonstrated that 300% enhanced photocurrent can be achieved in single SiNW-based solar cells^37^, compared with the bulk ones per unit volume. Similarly, in a single GaAs nanowire solar cell^4^, photocurrent of 180 mA cm^−2^ is obtained, which is more than one order of magnitude higher than the bulk counterpart. Here, we make efforts to quantitatively describe the size dependency of SiNW’s LHE-F, including LCE and LAE, which respectively characterize the antireflection performance and the photocurrent limitation can be generated in SiNW based devices. LCE (LAE) is calculated by integrating the extinction (absorption) efficiencies with the spectral photon flux density delivered by the sun, *AM1.5g*, in wavelength domain 0.2–1.1 μm; and its diameter and length dependencies are given in Fig. 4a,b, respectively.

Since the photon flux density in AM1.5 is wavelength dependent and meanwhile RW of SiNW is diameter sensitive, the diameter dependencies of LHE after integration (LHE-F) turn to have no clear physical significances. However, the integration has no effect on the length dependency of LHE, therefore LHE-F also shows perfect length dependency. From Fig. 4a it can be seen that LHE-F still first increases then decreases with diameter. So we also use two functions to describe LCE or LAE of SiNW, which are written as *Q*_*ext-int(w)-s*_, *Q*_*ext-int(w)-b*_, *Q*_*abs-int(w)-s*_ and *Q*_*abs-int(w)-b*_, respectively. They are all set as linear of length according to their perfect length dependency, but their order on diameter is chosen to be cubic with the criteria to obtain the best fitting results.

The fitting pictures and the quantitative expressions of these four functions are given in Fig. 4c,d. The R-squares, 0.999, 0.9994, 0.9992 and 0.9992, denote the perfect fittings. Furthermore, we have also calculated SiNW’s LHE-F by integrating the extinction (absorption) efficiencies with unit light intensity, which are signified by *Q*_*ext-int*_and *Q*_*abs-int*_, respectively. The regression process is given in the Supplementary Information, and the obtained equations and *R-squares* are given in Table 1.

Notably, there are also ridges in Fig. 4c,d, the figures of *Q*_*ext-int(w)*_and *Q*_*abs-int(w)*_. By letting *Q*_*ext-int(w)-s*_ equal to *Q*_*ext-int(w)-b*_, and *Q*_*abs-int(w)-s*_ equal to *Q*_*abs-int(w)-b*_, the expressions of these ridges are derived as


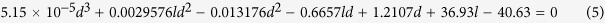


and


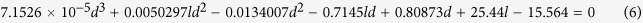


By them, the optimal diameters for SiNW of fixed length 0.5, 1.0, 1.5 and 2.0 μm are 97.2, 95.1, 99.2 and 101.5 nm in light concentration; and 68.3, 69.6, 71.1 and 71.5 nm in light absorption. These values are consistent with the best SiNW diameter in light-trapping, ~80 nm^37^,^50^.

To test the practicability of these equations, comparisons between their predicted results and those in previous reports, including experimental and theoretical results, are carried out. Since only the data for RW can be found, only comparisons for the RW are given, in Table 2.

On the whole, the established equations can give quite good predictions for RW. The errors between the predicted results and most of the reported (including experimental and theoretical) ones are smaller than 5% (about 25 nm). These errors are smaller than half of the half-width of Main peaks in Fig. 1. Such good consistence indicates the presented equations in this study are reliable and of practical significance. The big errors between the predicted results and the experimental ones in ref. 8 can be attributed to measuring errors of the SiNW’s diameter from SEM images, and the random incident angle (SiNW mats are disordered with random orientations). The errors between our predicted results and the theoretical ones in ref. 8 are much smaller: the index of silicon in the reference (3.5) is smaller than the real values (~3.8).

The expansibility of the established equations can be partly reflected by their good predictions on RW for SiNW with small lengths^31^,^39^ and arbitrary shapes^31^,^51^. Here, we systematically test the transverse (i.e. for thinner or thicker SiNWs) and longitudinal (i.e. for shorter or longer SiNWs) expansibility of the three group equations, by comparing their predicted results with the calculated ones using DDA.

**Figure 5:**
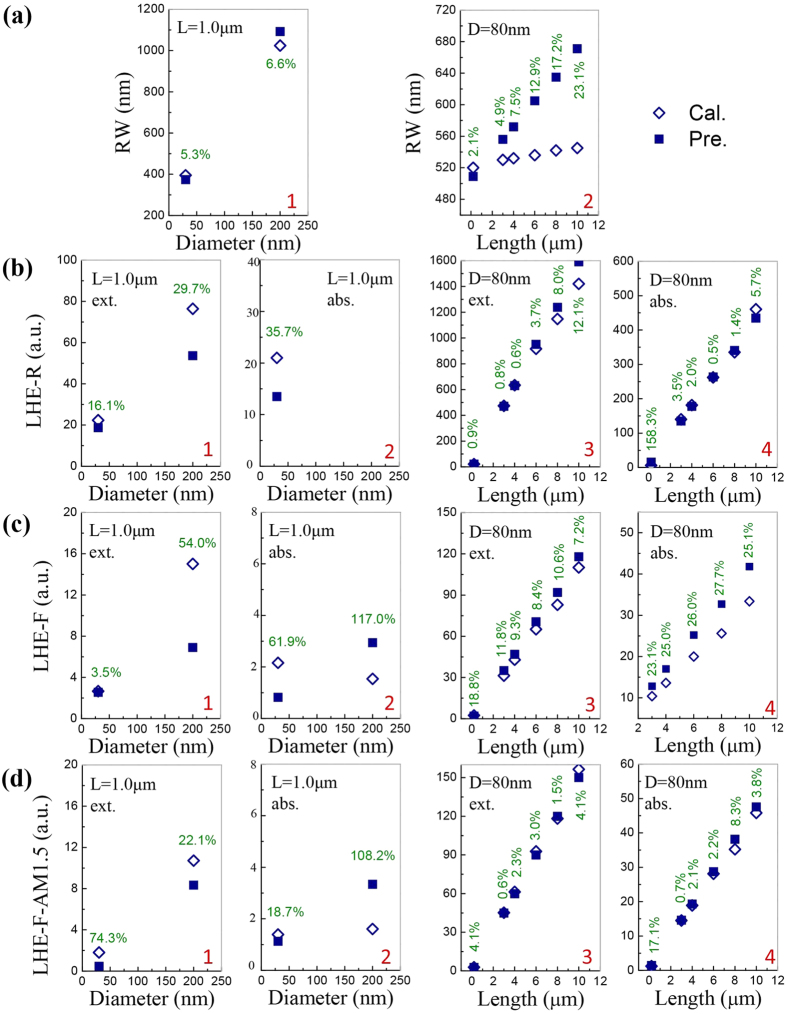
Comparisons between the predicted results using the equations and the calculated ones by DDA, for thinner, thicker, shorter and longer SiNW. (**a**) RW; (**b**) LHE-R; (**c**) LHE-F without weighting of *AM1.5g*; (**d**) LHE-F with weighting of *AM1.5g*. Relative errors are provided by percentage of expression.

At first, RW equations *λ*_*m*_, *λ*_*p1*_and *λ*_*p3*_ are tested. Figure 5a-1 shows the comparison between the predicted and calculated results of *λ*_*m*_, for thinner (30 nm) and thicker (200 nm) SiNW. It can be seen that the relative errors between the predicated and calculated results are ~5% and ~6.5%, corresponding absolute errors are ~25 nm and ~70 nm respectively. Referring Fig. 1, these absolute errors are less than the half-width of Main peaks. For *λ*_*p1*_and *λ*_*p3*_, the predicted values match with the calculated results, with errors 0.4–4.8%. In longitudinal direction, from Fig. 5a-2 it can be seen that, for shorter SiNW (0.2 μm), the predicted RW match very well with the calculated ones, with error 3.5%. However, for longer SiNW (3–10 μm), if the length of SiNW exceeds 3μm, the predicted *λ*_*m*_shows length dependent errors, which increase from 4.9% (3.0 μm) to 23.1% (10 μm). So, pertinent corrections should be made when using the equation of *λ*_*m*_to estimate the RW of SiNW with length greater than 3 μm. As a summary, the RW equations are of good transverse and longitudinal expansibility for SiNW with length from 0.2 to 3 μm.

Then, tests on LHE-R equations, *Q*_*ext-m*_ and *Q*_*abs-m*_, are carried out. For thinner and thicker SiNW, Fig. 5b-1 shows that *Q*_*ext-m*_ gives smaller predictions than calculated values, with errors 16–30%. Because it is unnecessary to give precise predictions of LHE-R for most applications of SiNW, such errors are acceptable. *Q*_*abs-m*_ can also give acceptable prediction for thinner (30 nm) SiNW, with error about 35%. While, the expansibility of *Q*_*abs-m*_for thicker (200 nm) SiNW cannot be tested due to the fact that the value of *Q*_*abs-m*_becomes very small because RW exceeds 1000 nm. And at this wavelength, silicon has a very small optical absorption coefficient. In longitudinal direction, Fig. 5b-3,b-4 shows that both the predicted *Q*_*ext-m*_and *Q*_*abs-m*_coincide quite well with the calculated results, no matter for shorter (0.2 μm) or longer (3.0–10.0 μm) SiNW. In conclusion, the LHE-R equations have very good transverse and longitudinal expansibility.

As a last step, the LHE-F equations are tested. As shown in Fig. 5c-1,c-2, equations *Q*_*ext-int*_ and *Q*_*abs-int*_can predict the LHE-F of SiNW with thinner and thicker diameter quite well. The prediction errors are 3.5–54% and 62–117% respectively. Meanwhile, they also have very good longitudinal expansibility (prediction errors 7.2–27.7%) as demonstrated in Fig. 5c-3,c-4. After *AM1.5g* is weighted, from Fig. 5d-1,d-2 it can be found that the equations *Q*_*ext-int(w)*_ and *Q*_*abs-int(w)*_ show similar transverse expansibility as *Q*_*ext-int*_ and *Q*_*abs-int*_, with errors 22–74% and 18–108% respectively. And from Fig. 5d-3,d-4 we can find that they are also of perfect longitudinal expansibility: the prediction errors for *Q*_*ext-int(w)*_ are less than 4.1%, and for *Q*_*abs-int(w)*_ range from 0.7% to 17.1%. In short, the LHE-F equations have good transverse and excellent longitudinal expansibility.

As a summary, we systematically investigate three important performance parameters for the PM properties of SiNW, including RW, LHE-R and LHE-F, by DDA simulations. Correspondingly, three-group equations are established for the prediction of these parameters of SiNW with given size. The first group of equations, *λ*_*m*_, *λ*_*p1*_and *λ*_*p3*,_ can provide exact predictions for RW of SiNW with error smaller than 1/2 of the half-width of the resonant peaks. They are of great importance for the design of SiNW-based devices with desired resonances. The second group contains eight expressions: *Q*_*ext-m-s*_, *Q*_*ext-m-b*_, *Q*_*abs-m-s*_, *Q*_*abs-m-b*_, *Q*_*ext-p1*_, *Q*_*abs-p1*_, *Q*_*ext-p3*_ and *Q*_*abs-p3*_, which describe the LCE and LAE of SiNW at different RW. They can be used to evaluate the best single frequency performance of SiNW-based photonic devices. The third group of equations, *Q*_*ext-int(w)-s*_, *Q*_*ext-int(w)-b*_, *Q*_*abs-int(w)-s*_, *Q*_*abs-int(w)-b*_, *Q*_*ext-int*_, *Q*_*abs-int-s*_and *Q*_*abs-int-b*_, describe the LCE and LAE of SiNW in full-spectrum under illumination of *AM1.5g* or unit light intensity. They are of special significance in photovoltaic fields as they can be used to calculate the limit photocurrent density generated in SiNW based solar cells. These three-group equations are not only practically reliable, but also expansible to predict the PM properties of SiNW with wider size range. Additionally, the optimal size of SiNW for light-harvesting is visually and quantitively presented: four extra equations are derived to predict the optimal size of SiNW in light-harvesting. This study provides a set of convenient analytical tools for the design and optimization of SiNW-based photonic devices.

## Methods

### Theoretical simulations

Extinction and absorption spectra are calculated using the DDA method. In the framework of DDA, firstly, the target is replaced by an array of point dipoles; then, the electromagnetic scattering problem for an incident light interacting with this array of point dipoles is solved by the iterative method. Therefore, the accuracy of DDA simulation depends on two factors: the interdipole spacing, d, and the error tolerance between two adjacent iterative steps, h. Herein, d and h are set as 3.3 nm and 1.0 × 10^−5^ respectively, which have been carefully tested in our previous works^44^,^45^.

## Additional Information

**How to cite this article**: Li, Y. *et al*. Exact comprehensive equations for the photon management properties of silicon nanowire. *Sci. Rep*. **6**, 24847; doi: 10.1038/srep24847 (2016).

## Supplementary Material

Supplementary Information

## Figures and Tables

**Table 1:** Equations for the PM properties of SiNW and corresponding R-squares.

**Equations**	**R-square**
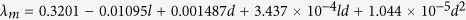	0.9981
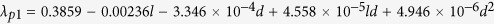	0.9971
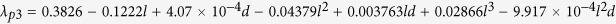	0.9998
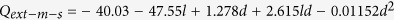	0.9981
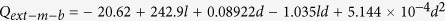	0.9991
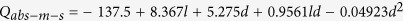	0.9982
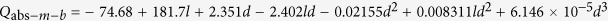	0.9926
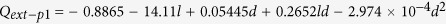	0.9984
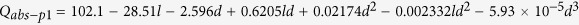	0.9964
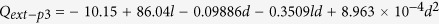	0.9979
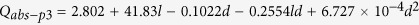	0.9795
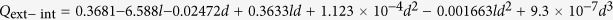	0.9989
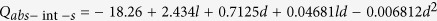	0.9928
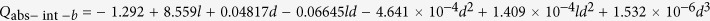	0.9978
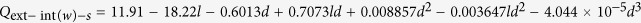	0.999
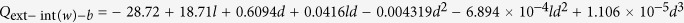	0.9994
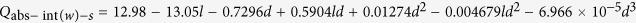	0.9992
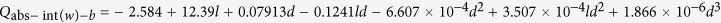	0.9992

**Table 2:** RW of SiNW being reported and predicted using our equations.

**Ref. No, measure angle, theoretical method**			**RW/nm**				
			**Size of SiNW**		**Reported**				**Err./%**	
	**L/μm**	**D/nm**			**Exp.**	**Theo.**	**Predicted**	**Exp.**			**Theo.**
15, θ = 5°	2.6	70	490		510	4.1	
8, θ = 90°, Mie theory		80	440	480	506	15.0	5.4
		60	390	420	447	14.6	6.4
		40	350	380	396	13.1	4.2
31, θ = 0°, FDTD	0.1	B82	440	430	442	0.5	2.8
		B124	525		554	5.5	
		B132	560		583	4.1	
37, θ = 20°, CLMT		100	540	540	573	6.1	6.1
39, FDTD	0.25	100		~560	579		3.3
52, θ = 5°	2	20~50	350~450		346~433	0.4	
51, Mie & FDTD		75 (*sph*)		~500	494		1.2
53, θ = 20°		77		470	496		5.5
		86		500	525		5.0
		107		575	599		4.2
		118		620	641		3.4

Abbreviations: FDTD, finite-difference time-domain; CLMT, coupled leaky mode theory; *sph*, nanosphere. Notes: For SiNW whose length is not provided in the literatures, in our prediction its length is set to be 0; Silicon nanostructure in ref. 31 is frustum, thus we use B to denote the base diameter and use the average diameter for prediction.
