# Supplementary material for: Exact comprehensive equations for the photon management properties of silicon nanowire
Source: Sci Rep. 2016 Apr 22;6:24847. doi: 10.1038/srep24847 (PMC4840328; doi:10.1038/srep24847)
Supplement: Supplementary Information [file srep24847-s1.pdf]

Correspondence and requests for materials should be addressed to M.C.L. (mcli@ncepu.edu.cn)

Exact comprehensive equations for the photon management properties of silicon nanowire

Yingfeng Li[1], Meicheng Li[1,2], Ruike Li[1], Pengfei Fu[1], Tai Wang[1], Younan Luo[1], Joseph Michel Mbengue[1] & Mwenya Trevor[1]

[1]State Key Laboratory of Alternate Electrical Power System with Renewable Energy Sources, North China Electric Power University, Beijing, 102206, China

[2]Chongqing Materials Research Institute, Chongqing, 400707, China

Assignment of the mode indices for RW of SiNW:

The four kinds of peaks, marked as Main peak, Peak1, Peak2 and Peak3 in Figure 1b, correspond to different modes supported by the SiNW. According to the leaky mode theory, if the wavelength of light (propagating in the nanowire) is longer than the cut-off wavelength of one specific mode, the light will be leaked out thus couple with (capture) the light surrounding the nanowire. For a specific mode, there will be a certain wavelength, some extent longer than the cut-off wavelength, where the nanowire can capture the most amount of light. This wavelength is the RW. The cut-off wavelengths can be calculated by letting the normalized frequency V equal to the cut-off parameters of every mode (Table S1). $V = \pi d / \lambda \sqrt{n^2 - 1}$, where d is the SiNW diameter, n is the refractive index of silicon, and λ is the wavelength of light. Taking the SiNW with diameter 100 nm as example, the obtained cut-off wavelengths are ~530 nm for TE_{01}, TM_{01} and HE_{21}, ~415 nm for EH_{11}, HE_{12} and HE_{21}, and ~380 nm for EH_{21} and HE_{41}.

Main peak has the longest wavelength thus it should correspond to the lowest-order modes. Therefore, it is no doubt to assign Main peak to the degeneracy of TE_{01}, TM_{01} and HE_{21}. This assignment can be confirmed by the fact that the electric field of the main peak follows the distribution of mode LP_{11} (a degenerate mode of TE_{01}, TM_{01} and HE_{21}), as given in the inset of Figure 1c. Similarly, Peak1 and Peak2 can be identified to the degeneracy of EH_{11}, HE_{12} and HE_{21}, and the degeneracy of EH_{21} and HE_{41}, respectively. And such identifications can be also confirmed by the consistence between the electric field distribution of them and of the LP_{21} (degenerate mode of EH_{11} and HE_{31}) and LP_{31} (degenerate mode of EH_{21} and HE_{41}) modes, respectively.

Cut-off parameters	2.405	2.42	3.83	3.86	5.14	5.16
Mode index	TE_{01}<br>TM_{01}	HE_{21}	HE_{12}<br>EH_{11}	HE_{31}	EH_{21}	HE_{41}

Table S1. Cut-off parameters of the first few guided modes

Peak3 can't be assigned to mode LP_{21} since its wavelength, 517 nm, is much longer than the cut-off wavelength of mode LP_{21}, 415 nm. Besides, i) its electric field distribution shows obvious longitudinal oscillation pattern Figure 1c; ii) it becomes weaker until disappears as the length of SiNW increases, as shown in Figure S1, which reflects the variation of the extinction and absorption curves for SiNWs with increased length. What's shown in the insets are Peak3, appearing when the diameters greater than 90 nm. It can be seen that, with the length increases, Peak3 become more and more unobvious, even vanish for the SiNW with diameter 90 and 100 nm. Taking into account it can only occurs when the diameter of the SiNW greater than 90 nm, this phenomenon indicates that Peak3 may come from the limitation on the length direction, and should be greatly related to the draw ratio of the SiNW.

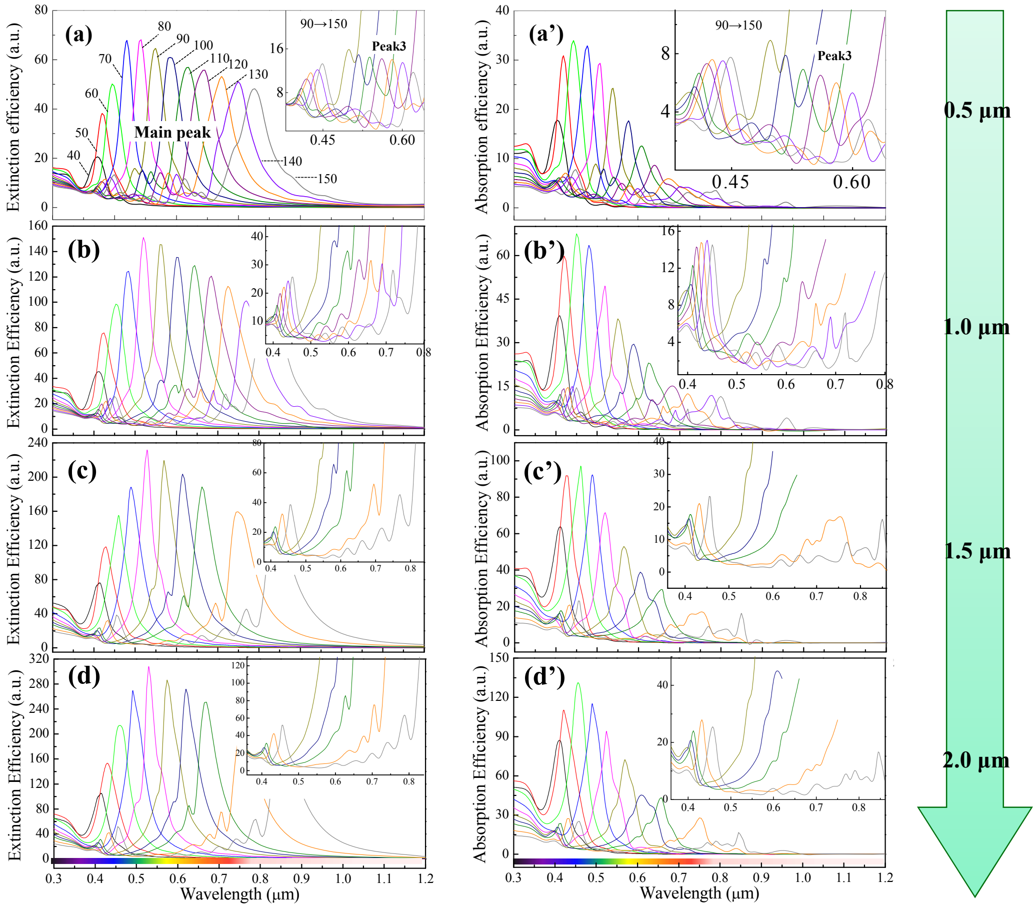

Figure S1. The variation of the extinction and absorption curves for SiNWs with increased length. The labels with and without ['] denote the extinction and absorption curves, respectively. (a-d) correspond to lengths 0.5, 1.0, 1.5 and 2.0 µm, respectively.

Relationship between RW and the cut-off wavelength of SiNW:

From the waveguide theory, the cut-off wavelength is the critical wavelength where the mode can be leaked out.

At the cut-off wavelength, the light propagating in the nanowire **starts to** be leaked out (very little light can be leaked out), to couple with the light surround the nanowire (very little light can be coupled within the NW). With the wavelength increases, more light can be leaked out, and correspondingly more light can be coupled within the Si NW, till a **peak value** which corresponds

to the **resonance wavelength.** While, when the wavelength is much longer than the cut-off wavelength, the leaky mode cannot be supported again. Therefore, the actual resonance wavelength should be some greater than the cut-off wavelength.

We have compared RW of SiNWs (with fixed length, 1μm, and various diameter 40, 60, 80, 100, 120 and 140 nm) calculated by the DDA simulations and those by the equation $V = \pi d / \lambda \sqrt{n^2 - 1}$. The values calculated by the equation exactly coincide with the leftmost wavelength of the resonance peaks. This effectively verified the above explanations.

The fitting pictures for RW of peak1 and peak3.

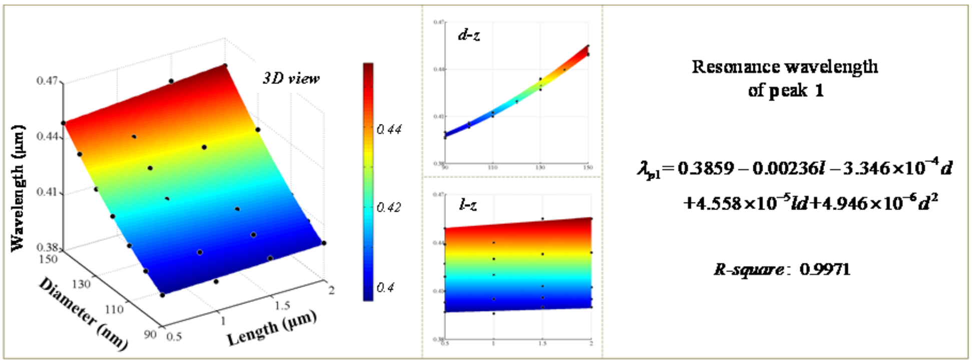

Figure S2. Fitting picture for RW of peak1. Original data (black spheres) of RW at peak1, and the interpolated surface. The *3D view*, *d-z*, *l-z* side views of the fitting picture, and the polynomial with *R-square* are given.

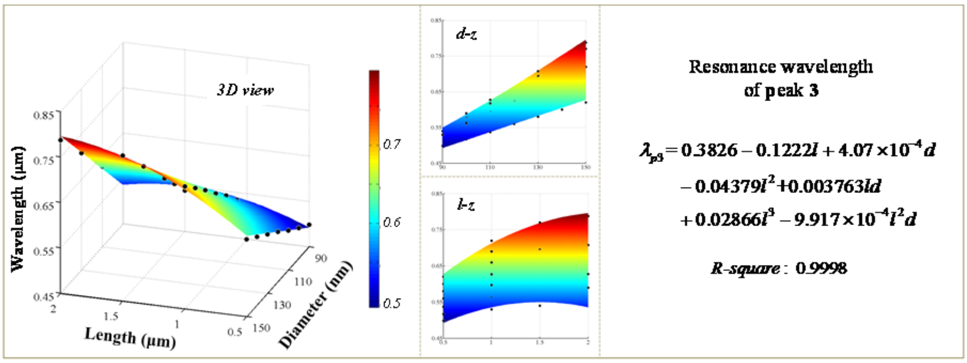

Figure S3. Fitting picture for RW of peak3. Original data (black spheres) of RW at peak3, and the interpolated surface. The *3D view*, *d-z*, *l-z* side views of the fitting picture, and the polynomial with *R-square* are given.

Fitting pictures for the extinction and absorption intensities at peak1 and peak3.

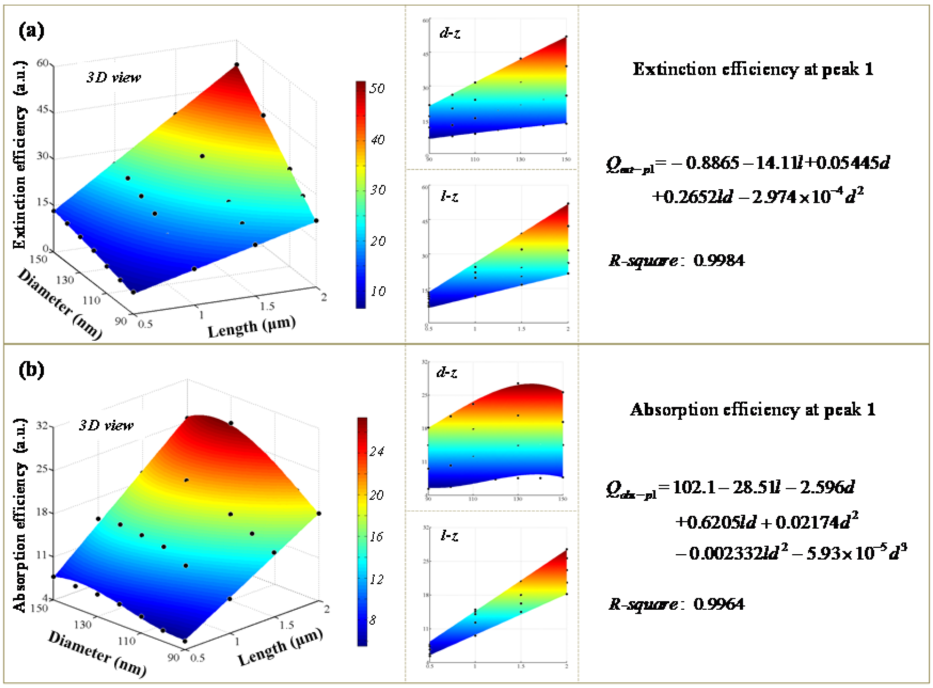

Figure S4. Fitting pictures for the extinction and absorption intensities at peak1. Original data (black spheres) of the (a) extinction and (b) absorption intensities of peak1, and the interpolated surface. The *3D view*, *d-z*, *l-z* side views, and the polynomial with *R-square* are given.

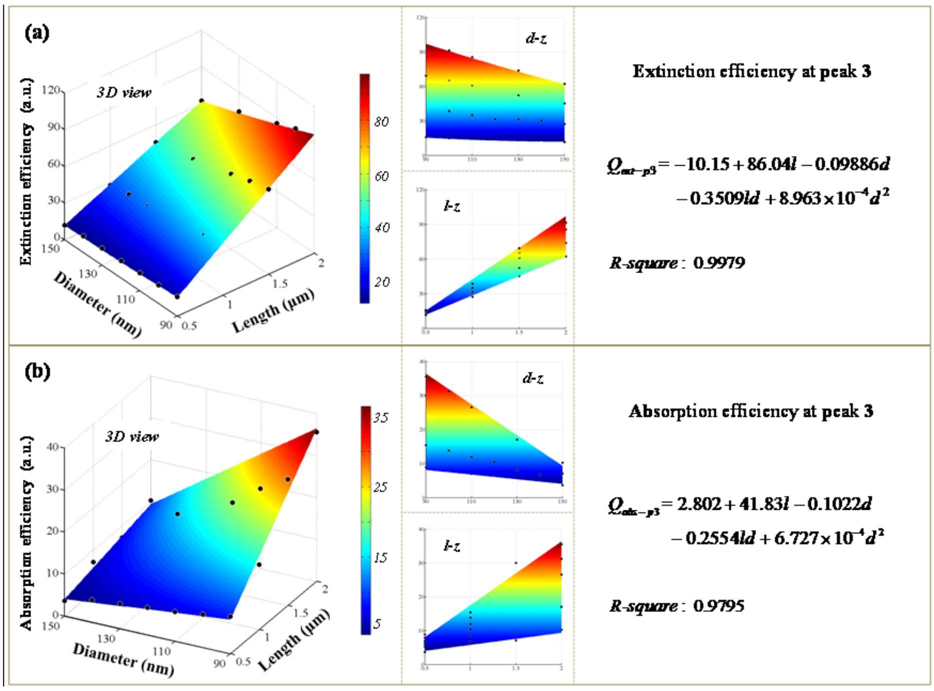

Figure S5. Fitting pictures for the extinction and absorption intensities at peak3. Original data (black spheres) of the (a) extinction and (b) absorption intensities of peak3, and the interpolated surface. The *3D view*, *d-z*, *l-z* side views and the polynomial with *R-square* are given.

Regression process of the equations for the integrated extinction and absorption intensities in full-spectrum with unit light intensity.

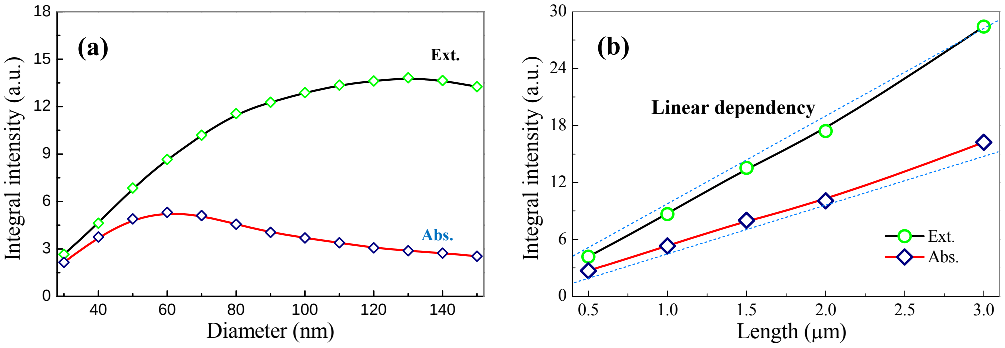

Figure S6. Size dependency of LHM-F without weighting of *AM1.5g*. a) Diameter dependency; b) length dependency.

The integrated extinction and absorption efficiencies are signified by $Q_{ext\text{-}int}$ and $Q_{abs\text{-}int}$, respectively. **Figure S6a** shows that both of them firstly increase and then decrease with increased diameter. However, when we try to fit the data of the integrated extinction efficiencies, we found that using one cubic function of the diameter is better than using two separated quadratic functions, in describing total variation in the data. Therefore, the practical function used is a polynomial owning cubic term of the diameter. Figure S6b shows that both of $Q_{ext\text{-}int}$ and $Q_{abs\text{-}int}$, show well linearity with length.

By regression method, the final equation to describe the integrated extinction intensity (Figure S7a) of SiNW is

$$Q_{\text{ext-int}} = 0.3681\text{-}6.588l\text{-}0.02472d + 0.3633ld + 1.123\times10^{-4}d^2 - 0.001663ld^2 + 9.3\times10^{-7}d^3$$

with correlation index *R-squares=0.9989*, which reflects the good fitness.

For the integrated absorption intensity (Figure S7b), two functions can give very good fitting results to the original data. We attribute the dependent rules of $Q_{abs\text{-}int}$ before and after the points to

be quadratic and cubic, respectively. The obtained equations are

$$Q_{abs-\text{int}-s} = -18.26 + 2.434l + 0.7125d + 0.04681ld - 0.006812d^2$$

and

$$Q_{\text{abs-int-b}} = -1.292 + 8.559l + 0.04817d - 0.06645ld - 4.641\times10^{-4}d^2 + 1.409\times10^{-4}ld^2 + 1.532\times10^{-6}d^3 .$$

And their correlation indexes R-square are 0.9928 and 0.9978, respectively. The dash black line on the d-z side views in Figure S7b denotes the optimal size of SiNW to get the maximum integrated light-absorption ability.

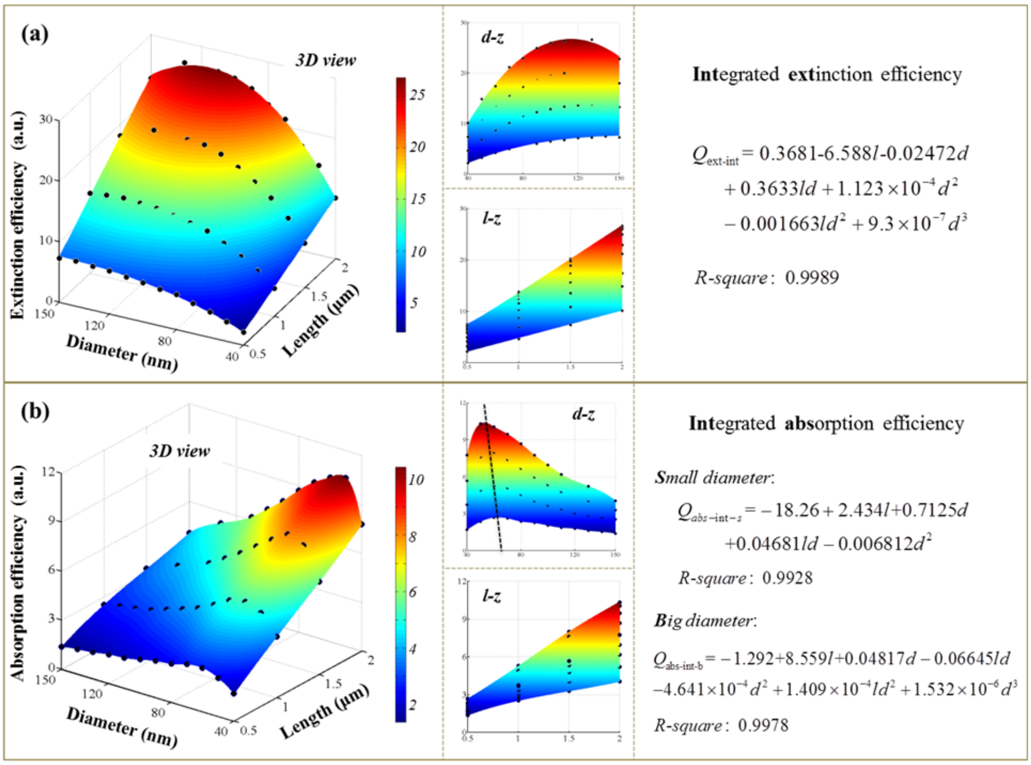

Figure S7. Fitting pictures for LHE-F without weighting of AM1.5. Original data (black spheres) of the integrated (a) extinction and (b) absorption intensities, and the corresponding interpolated surface. The *3D view*, *d-z*, *l-z* side views and the fitted polynomial with *R-square* are provided.
